# Contraceptive utilization among new exotic dancers: a cross-sectional study

**DOI:** 10.1186/s12954-018-0261-7

**Published:** 2018-11-12

**Authors:** Mishka Terplan, Caitlin E. Martin, Jennifer Nail, Susan G. Sherman

**Affiliations:** 1 Departments of Obstetrics and Gynecology and Psychiatry, Virginia Commonwealth University, PO Box 980268, Richmond, VA 23298 USA; 2 Department of Obstetrics and Gynecology, Virginia Commonwealth University, PO Box 980034, Richmond, VA 23298 USA; 3 Department of Mental Health, Bloomberg School of Public Health, The Johns Hopkins University, 550 N. Broadway, Baltimore, MD 22205 USA; 4 Department of Health Behavior and Society, Bloomberg School of Public Health, The Johns Hopkins University, 624 N. Broadway, Hampton House 749, Baltimore, MD 21205 USA

**Keywords:** Contraception, Exotic dancer, Harm reduction, Substance use disorder

## Abstract

**Background:**

Female exotic dancers are a population at high risk of unintended pregnancy. The objective of this study is to describe the reproductive health needs and contraceptive utilization of exotic dancers.

**Methods:**

New exotic dancers (< 6 months dancing) from 26 clubs in Baltimore City/County completed a one-time survey.

**Results:**

Of 117 participants, 96 (82%) had current contraceptive need. The mean age was 24 years, and 55% were black. Sex work (45%), alcohol use disorder (73%), illicit (44%; e.g., heroin, crack, cocaine), and injection drug use (8%) were common. The majority (66%) reported contraception use in the prior 6 months. Condoms were reported by 46% whereas 45% reported non-barrier methods, most commonly hormonal injection. Consistent condom use was rare (3%), and only 11% used a long-acting reversible method.

**Conclusions:**

Despite their unique reproductive health vulnerabilities, female exotic dancers have unmet contraceptive needs. Targeted harm reduction strategies are needed to fill this gap.

## Background

Female exotic dancers experience a unique set of vulnerabilities, owing greatly to their work environment [1]. Drug use, sex exchange (for money or drugs), and multiple sex partners are common [2]. Consequently, the small body of literature on this population focuses largely on infectious disease, specifically emphasizing HIV prevention and condom promotion, with little attention to their reproductive health [3]. Little is known about their non-condom contraceptive use in the USA.

Given the high rate of unintended pregnancies [4] in the USA and the unknown reproductive needs of exotic dancers [5], we report on a contraceptive method choice survey that was embedded in a larger study.

## Methods

Data were drawn from a baseline survey investigating the role of the exotic dance club environment on the HIV/STI (sexually transmitted infection) risk profile of dancers over time. Exotic dance clubs in Baltimore City and County, MD were purposively recruited between May and October 2013. Of the 26 clubs approached, 22 consented to participate. Dancers within these clubs were approached by research staff. Inclusion criteria included age 18 or older, dancing for 12 months or less, and on three or more occasions in the past month. Exclusion criteria included appearing cognitively impaired or intoxicated. Of 144 eligible women identified, 117 (81%) provided informed consent and completed the survey using Audio Computer-Assisted Self-Interview (ACASI) software. The survey lasted approximately 1 hour and was performed in a range of confidential venues (e.g., exotic dance club, private houses, restaurants, cafes, cars). Demographic (e.g., age, ethnicity), dancing duration, sex work (e.g., exchanging sex for money, drug, food, or a place to stay), past 6 month substance use, reproductive health, and health care utilization data were collected. Unstable housing was defined as not owning or renting one’s own home. Women with current contraceptive need were defined as non-pregnant participants who reported being heterosexually active in the prior 6 months. Contraceptive method choice was captured for the prior 6 months and included sterilization, hormonal methods, condoms, and emergency contraception. Participants received an $80 pre-paid debit card.

Descriptive analyses (STATA, Version 13) were employed to describe contraceptive need, utilization, and method choice. Results are reported by population and frequency. Given the low reported non-barrier contraceptive utilization, additional analysis was not warranted.

## Results

One hundred and two (87%) participants were sexually active in prior 6 months, of whom 98 (96%) had at least 1 male partner and 2 were pregnant. Hence, 96 women were identified as having contraceptive need and included in subsequent analyses.

The mean age of the study population was 24 (range 18–43 years) with 55% self-identified as black, 39% white, and 6% other race. Most (89%) had completed high school, and 42% had attended at least some college. Unstable housing was reported by 42%, and 45% reported a history of sex work. Substance use was common with 44% reporting past 6 month illicit drug use (e.g., heroin, crack, cocaine), 73% meeting criteria for an alcohol use disorder, 8% reporting past 6 month injection drug use, and 15% reporting lifetime receipt of substance use disorder treatment. Among those with prior pregnancies (*n* = 75; 78%), the mean reported number of pregnancies was 2.4 (range 1–7). Of these, 33% had a prior abortion, and an additional 14% reported having had two or more. Six percent reported their last STI test was positive. Most had health insurance (82%) and had accessed health care in prior 12 months (81%), but 47% reported experiencing stigma from health care providers (Table 1).

**Table 1:** Demographics, health service, and contraception use among female exotic dancers with current contraceptive need (*N* = 96)

Demographics	*N* (%) or mean (range)
Mean age in years	24 (17.7–42.9)
Race	
White	37 (39%)
Black	53 (55%)
Other	6 (6%)
Married or “in a serious relationship”	27 (28%)
Prior pregnancy	75 (78%)
Have children at home	38 (40%)
Education	
Did not complete high school	11 (11%)
High school diploma or GED	45 (47%)
Some college or above	40 (42%)
Living in unstable housing	41 (42%)
General and reproductive/sexual health history	
History of sex work	43 (45%)
Prior abortion	35 (33%)
2 or more prior abortions	13 (14%)
Sexually active with 4 or more male partners in past 6 months	27 (28%)
Last HIV/STI test positive	6 (6%)
Alcohol and drug use	
Active alcohol use disorder	70 (73%)
Illicit drug use in past 6 months (e.g., heroin, crack, cocaine)	42 (44%)
Past 6 month injection drug use	8 (8%)
Ever received substance use treatment	14 (15%)
Healthcare use and barriers to seeking services	
Has health insurance	79 (82%)
Last saw healthcare provider > 12 months ago	18 (19%)
Recently sought reproductive health service in emergency department	14 (18%)
Reports stigma seeking healthcare	18 (47%)
Wanted to see healthcare provider but was not able to do so	13 (14%)
Past 6-month contraceptive use	
Any contraception	66 (66%)
Any non-barrier method	43 (45%)
Sterilization	2 (5%)
Oral contraceptive pills	12 (29%)
Injection	16 (39%)
Implant	3 (7%)
Intrauterine device (IUD)	8 (20%)
Emergency contraception	5 (12%)
Any barrier method	44 (46%)
Male condoms	44 (100%)
Female condoms	3 (14%)
Consistent condom use	3 (14%)
Dual protection	25 (26%)
Other method	1 (1%)

The majority (*n* = 66; 66%) reported any contraception use, with 46% using male or female condoms and 45% using a non-barrier method. The most common non-barrier methods used included hormonal injections (*n* = 16) and oral contraceptive pills (*n* = 12). Few were using a long-acting reversible contraceptive (LARC) method such as an intrauterine device (IUD; *n* = 8) or hormonal implant (*n* = 3). Dual method use (condoms in addition to other more effective contraceptive methods) was reported by 26%. However, consistent condom use, defined as using condoms consistently (“always” during any vaginal sex), was rare (3%; Table 1).

## Discussion

Among women new to exotic dancing, we found a large burden of unmet contraceptive need. Although many women reported using condoms and a quarter dual method use, LARC and consistent condom use were rare.

A small body of research related to exotic dancers exists, but it is almost exclusively international and focused on female sex work that occurs within exotic dance clubs. Martin et al. reported on contraceptive method choice in Russian sex workers. Among women with contraceptive need, almost all reported use of barrier methods, and one-third reported non-barrier methods, the most common being oral contraceptives followed by intrauterine devices. Nonetheless, 10.6% reported consistent condom use and 5.5% dual protection [6]. In a study of street-based female sex workers in Canada, two-thirds reported condom use with clients, and one-third reported female-controlled contraceptives (excluding condoms but including hysterectomy), the most common being tubal ligation followed by injectables [5]. In our study, we found similar rates of non-barrier method use but much lower rates of consistent condom use in our study.

In the USA, almost half of all pregnancies are unplanned, of which almost 40% end in abortion; despite recent trends towards decreased incidence of unintended pregnancy, disparities in rates still exist [4]. Among exotic dancers, low rates of both consistent condom and effective contraception use lead to higher risk of HIV and STI acquisition, unplanned pregnancy, and possibly abortion. This risk is compounded by structural factors inherent in the exotic dance industry, including economic vulnerability, risky work environments, and social networks which facilitate sexual risk behavior [7].

This study has several limitations. Data are self-reported and therefore subject to social desirability bias particularly for drug use, sexual behaviors, and prior abortions which could be more prevalent than reported, although use of ACASI should decrease this bias. In terms of contraceptive utilization, women tend to exaggerate method adherence (particularly to contraceptive pills) [8]. Hence, the true contraceptive prevalence may be even lower. Also, we did not assess pregnancy desires, and thus all women cannot be assumed to not desire pregnancy which may overinflate the calculated unmet contraceptive need. However, female exotic dancers by the nature of the risk environment in which they work generally do have a need for reliable contraception as sex work and sexual violence are prevalent [1]. Lastly, the sample’s small size precluded multivariate analyses. Strengths of the study include a high participation rate and the fact that this is the first study to examine contraceptive utilization among exotic dancers in the USA.

## Conclusions

Female exotic dancers are at high risk for unintended pregnancy given the risk environment where they work. For all women, access to quality contraceptive information and services are central to women’s ability to control their reproductive lives and ultimately for sustaining reproductive justice [9]. Public health interventions in this realm, though, need to be tailored to the specific needs of a population to be effective. For example, harm reduction approaches such as street-based outreach and mobile services (e.g., needle exchange, condoms) have been shown to be successful in both HIV prevention and contraceptive adherence in this population [10]. Similar strategies specific for female exotic dancers should be pursued to address the unmet reproductive and sexual health needs of exotic dancers.
